# The Impact of Repeat Hospitalizations on Hospitalization Rates for Selected Conditions Among Adults With and Without Diabetes, 12 US States, 2011

**DOI:** 10.5888/pcd12.150274

**Published:** 2015-11-19

**Authors:** Stephanie M. Benjamin, Jing Wang, Linda S. Geiss, Theodore J. Thompson, Edward W. Gregg

**Affiliations:** Author Affiliations: Jing Wang, Linda S. Geiss, Theodore J. Thompson, Edward W. Gregg, Division of Diabetes Translation, Centers for Disease Control and Prevention, Atlanta, Georgia.

## Abstract

**Introduction:**

Hospitalization data typically cannot be used to estimate the number of individuals hospitalized annually because individuals are not tracked over time and may be hospitalized multiple times annually. We examined the impact of repeat hospitalizations on hospitalization rates for various conditions and on comparison of rates by diabetes status.

**Methods:**

We analyzed hospitalization data for which repeat hospitalizations could be distinguished among adults aged 18 or older from 12 states using the 2011 Agency for Healthcare Research and Quality’s State Inpatient Databases. The Behavioral Risk Factor Surveillance System was used to estimate the number of adults with and without diagnosed diabetes in each state (denominator). We calculated percentage increases due to repeat hospitalizations in rates and compared the ratio of diabetes with non-diabetes rates while excluding and including repeat hospitalizations.

**Results:**

Regardless of diabetes status, hospitalization rates were considerably higher when repeat hospitalizations within a calendar year were included. The magnitude of the differences varied by condition. Among adults with diabetes, rates ranged from 13.0% higher for stroke to 41.6% higher for heart failure; for adults without diabetes, these rates ranged from 9.5% higher for stroke to 25.2% higher for heart failure. Ratios of diabetes versus non-diabetes rates were similar with and without repeat hospitalizations.

**Conclusion:**

Hospitalization rates that include repeat hospitalizations overestimate rates in individuals, and this overestimation is especially pronounced for some causes. However, the inclusion of repeat hospitalizations for common diabetes-related causes had little impact on rates by diabetes status.

## Introduction

Hospitalization discharge data are often used to monitor diabetes-related outcomes, such as amputation and heart attacks, and to compare the risk for these outcomes among adults with and without diabetes (1–4). However, most hospital discharge data sources do not provide the information needed to separate individuals from events (5,6). For this reason, annual hospitalization rates are typically calculated by using the total number of hospitalizations (including repeat hospitalizations) instead of the number of individuals hospitalized (excluding repeat hospitalizations) in the calendar year. Although the inclusion of repeat hospitalizations in rates may provide a measure of service utilization, their inclusion could bias estimates of disease incidence and complicate analysis of disease trends, especially for chronic diseases.

The purpose of our study was to examine the impact of repeat hospitalizations on the magnitude of hospitalization rates for common diabetes-related outcomes or comorbidities and on the comparison of hospitalization rates for these causes by diabetes status.

## Methods

We analyzed 2011 hospitalization data for adults aged 18 years or older from the State Inpatient Databases (SID) of the Agency for Healthcare Research and Quality (AHRQ) (7). SID contains information on inpatient discharges from hospitals in a given state, regardless of payer. The participation of 48 states in SID enables it to capture approximately 97% of all US community hospital discharges. SID has more than 100 clinical and nonclinical variables, including principal and secondary diagnoses and procedures, admission and discharge status, patient demographic characteristics, expected payment source, total charges, and length of stay. A detailed description of the data are available at www.hcup-us.ahrq.gov/sidoverview.jsp.

We analyzed data only from 12 states (Arkansas, California, Florida, Hawaii, Iowa, Massachusetts, Mississippi, Nebraska, New Mexico, New York, Vermont, and Washington) whose discharge data distinguished repeat hospitalizations among individuals. The hospitalization data in the 12 states provided synthetic identification numbers (IDs) to link persons across events (ie, hospitalizations, emergency department visits, and ambulatory surgeries). The percentage of missing synthetic IDs for patients aged 18 to 64 was 2.1% for Arizona, 11.7% for California, 4% for Florida, 30.9% for Iowa, 6.5% for Massachusetts, 7.6% for Mississippi, none for Nebraska, 2.4% for New Mexico, 4% for New York, 7.3% for Utah, 13% for Vermont, and none for Washington (8). The percentage missing synthetic IDs for patients aged 65 years or older was less than 0.4% for Arizona, 2.6% for California, 1.2% for Florida, 19.1% for Iowa, 2.3% for Massachusetts, 8.4% for Mississippi, none for Nebraska, 2.5% for New Mexico, less than 0.5% for New York, 2.4% for Utah, 13.8% for Vermont, and none for Washington (8). For records missing IDs, we multiply imputed whether a case was a repeated hospitalization based on the person’s age, sex, race/ethnicity, insurance, median income category of residence zip code, and comorbidity score (9,10), using fully conditional specification methods. A sensitivity analysis showed no significant differences between estimates that used imputed data and estimates based on deleting missing data.

We excluded noncommunity or rehabilitation hospitals on the basis of information from the American Hospital Association’s Annual Survey Database. In our analyses, we defined repeat hospitalizations as more than 1 hospitalization of the same person for the same condition or discharge diagnosis, in a given calendar year. This concept is different from that of readmission, which typically uses a 30-day period and is used to examine quality of care.

Hospitalization rates, with and without repeat hospitalizations for selected causes, were calculated for people with and without diabetes. We used the first-listed discharge diagnosis code to identify hospitalizations due to the following causes: major cardiovascular disease (*International Classification of Diseases, Clinical Modification, 9th Revision* [ICD-9-CM] code of 390–434, 436–448), ischemic heart disease (ICD-9-CM code of 410–414,429.2), acute myocardial infarction (ICD-9-CM code of 410), heart failure (ICD-9-CM code of 428), stroke (ICD-9-CM code of 430–434, 436–438), peripheral arterial disease (ICD-9-CM code of 250.7, 440.2, 442.3, 443.81, 443.89, 443.9, 444.22), and lower extremity ulcer/inflammation (ICD-9-CM code of 454, 707.1, 680. 6–680.7, 681.1, 682.6–682.7, 711.05–711.07, 730.05–730.07, 730.15–730.17, 730.25–730.27, 730.35–730.37, 730.85–730.87, 730.95–730.97, 785.4). Lower extremity amputations were identified by using ICD-9-CM procedure code of 84.1 on the basis of all listed procedures, excluding traumatic amputation based on diagnosis code of 895–897 in any listed diagnosis. Among patients with diabetes, we identified emergency department visits for hypoglycemia by using a first-listed diagnostic code of 251.0, 251.1, 251.2, or 962.3; or a first-listed diagnostic code of 250.8 that was not accompanied by any of the following codes: 259.8, 272.7, 681, 682, 686.9, 707.1, 707.8, 707.9, 709.3, 730.0, 730.1, 730.2, or 731.8. Visits for hyperglycemic crisis were defined as those with 250.1 or 250.2 as the first-listed diagnosis. Discharges with any diagnosis code for diabetes (ICD-9-CM 250.x) on any patient hospitalization were considered diabetes-related.

To determine the number of patients with and without diabetes to be used as the denominator for the calculation of hospitalization rates, we used data from the 2011 Behavioral Risk Factor Surveillance System (BRFSS). BRFSS is a collaborative project of the Centers for Disease Control and Prevention (CDC) and US states and territories that collects information on health behaviors and conditions using state-based, ongoing, random-digit–dialed telephone surveys of noninstitutionalized US civilian adults aged 18 or older (n = 144,433 for the 12 states). The median response rate of all states in 2011 was 49.7% (11). The prevalence of diagnosed diabetes was calculated as the percentage of the population answering yes to the question “Have you ever been told by a doctor that you have diabetes?” Women who had been told that they had diabetes only during pregnancy and respondents told they had prediabetes or borderline diabetes were not considered to have diabetes.

We calculated rates of hospitalization (with and without repeat hospitalizations) and calculated rate ratios by dividing rates for diabetes by non-diabetic rates. All rates were age-adjusted to the 2000 US census population using age groups 18 to 44 years, 45 to 64 years, 65 to 74 years, and 75 years or older. Because hospitalization data are a complete enumeration of events, we do not associate any sampling error with them; the confidence intervals (CIs) of the rates reflect the sampling error of the denominators derived from BRFSS and the variance from multiple imputation. We examined the extent to which age-adjusted rates were overestimated by calculating percentage increases in rates caused by including repeat hospitalizations of individuals. The percentage increases in rates simplify to a form that includes only complete counts of hospitalizations and the variance induced by our multiple imputation methods was so small that we ignored it; therefore, the variances of percentage increases are zero.

All analyses were conducted by using SAS 9.1.3 (SAS Institute, Inc) and SUDAAN 11.0.1 (RTI International) to account for the complex sample designs of BRFSS. We used the delta method to estimate the standard errors associated with hospitalization rates and rate ratios (12).

## Results

Our study included 10,384,306 hospital discharges from 12 states in 2011. Regardless of diabetes status, repeat hospitalizations occurred for all examined causes, although their impact on rates varied by cause (Table). Compared with rates excluding repeats, rates including repeat hospitalizations for adults with diabetes ranged from 13.0% higher for stroke to 41.6% higher for heart failure; for adults without diabetes, rates including repeats were 9.5% higher for stroke to 25.2% higher for heart failure (Table). In general, for each state (Appendix) and overall, increases in rates caused by including repeat hospitalizations were higher for adults with diabetes than for those without diabetes. For heart failure and cardiovascular disease, the increase in rates due to repeat hospitalizations were approximately 10 percentage points higher for patients with diabetes compared with patients without diabetes. Hospitalizations due to hypoglycemia and hyperglycemic crisis, which are specific to patients with diabetes, also increased hospitalization rates when repeat hospitalizations were included. For hypoglycemia, the rate of increase was 9.9%, and for hyperglycemic crisis it was 50.9%.

**Table:** Cause-Specific Hospitalization Rates^a^, Including and Excluding Repeat Hospitalizations, Among Adults With and Without Diabetes, 12 States^b^, 2011

Conditions	Repeat Hospitalizations Included^c^	Hospitalization Rates (per 100 population) (95% CI)			
		Diabetes	Non-diabetes	Total	Rate Ratio (Diabetes/Non-diabetes)
Acute myocardial infarction	Yes	0.63 (0.60–0.65)	0.16 (0.16–0.16)	0.22 (0.22–0.22)	3.87 (3.73–4.02)
	No	0.54 (0.52–0.56)	0.15 (0.14–0.15)	0.20 (0.19–0.20)	3.68 (3.54–3.82)
	% Increase	16.02	10.20	12.52	—

Heart failure	Yes	1.14 (1.10–1.18)	0.21 (0.21–0.21)	0.34 (0.33–0.34)	5.45 (5.23–5.67)
	No	0.81 (0.78–0.84)	0.17 (0.16–0.17)	0.26 (0.25–0.26)	4.82 (4.63–5.01)
	% Increase	41.60	25.20	31.39	—

Cardiovascular disease	Yes	4.78 (4.61–4.96)	1.30 (1.29–1.32)	1.75 (1.73–1.77)	3.67 (3.53–3.81)
	No	3.44 (3.32–3.57)	1.07 (1.05–1.08)	1.37 (1.35–1.39)	3.22 (3.10–3.35)
	% Increase	38.98	22.07	27.63	—

Ischemic heart disease	Yes	1.41 (1.36–1.46)	0.33 (0.32–0.33)	0.47 (0.46–0.47)	4.29 (4.13–4.45)
	No	1.15 (1.11–1.19)	0.29 (0.28–0.29)	0.40 (0.39–0.40)	4.01 (3.86–4.16)
	% Increase	22.34	14.38	17.31	—

Stroke	Yes	0.65 (0.62–0.67)	0.22 (0.22–0.22)	0.28 (0.27–0.28)	2.93 (2.83–3.04)
	No	0.57 (0.55–0.59)	0.20 (0.20–0.20)	0.25 (0.25–0.25)	2.84 (2.74–2.95)
	% Increase	13.02	9.48	10.56	—

Lower extremity amputation	Yes	0.34 (0.33–0.36)	0.01 (0.01–0.01)	0.05 (0.05–0.05)	29.6 (28.03–31.10)
	No	0.29 (0.28–0.31)	0.01 (0.01–0.01)	0.04 (0.04–0.04)	27.36 (25.94–28.78)
	% Increase	18.04	9.24	16.45	—

Peripheral arterial disease	Yes	0.31 (0.30–0.33)	0.04 (0.04–0.04)	0.07 (0.07–0.07)	8.68 (8.35–9.01)
	No	0.25 (0.24–0.26)	0.03 (0.03–0.03)	0.06 (0.06–0.06)	8.02 (7.71–8.32)
	% Increase	24.93	15.37	21.55	—

Lower extremity ulcer	Yes	0.62 (0.59–0.66)	0.11 (0.11–0.12)	0.16 (0.16–0.16)	5.42 (5.11–5.72)
	No	0.53 (0.50–0.56)	0.10 (0.10–0.10)	0.14 (0.14–0.14)	5.23 (4.93–5.52)
	% Increase	17.31	13.20	14.46	—

Hypoglycemia	Yes	0.31 (0.29–0.32)	—	—	—
	No	0.28 (0.26–0.29)	—	—	—
	% Increase	9.93	—	—	—

Hyperglycemic crisis	Yes	1.67 (1.50–1.84)	—	—	—
	No	1.11 (1.00–1.22)	—	—	—
	% Increase	50.85	—	—	—

Abbreviations: —, does not apply; CI, confidence interval.

a Age-adjusted for adults aged 18–44, 45–64, 65–74, ≥75, based on the 2000 census.

b The 12 states are Arkansas, California, Florida, Hawaii, Iowa, Massachusetts, Mississippi, Nebraska, New Mexico, New York, Vermont, and Washington.

c Percentage increase is the increase due to including repeat hospitalizations.

Regardless of cause of hospitalization, rates for adults with diabetes were considerably higher than rates for adults without diabetes (Table). Ratios of diabetes versus non-diabetes rates were similar regardless of whether repeat hospitalizations were included. Overall, rate ratios including repeats ranged from 2.9 (95% CI, 2.8–3.0) for stroke to 29.6 (95% CI, 28.0–31.1) for lower extremity amputation (Table). Rate ratios excluding repeats ranged from 2.8 (95% CI, 2.7–3.0) for stroke to 27.4 (95% CI, 25.9–28.8) for lower extremity amputation. At the state level, rate ratios that included and excluded repeat hospitalizations were also similar (Appendix).

## Discussion

Using hospitalization data from 12 states, this study demonstrated that repeat hospitalizations for the same causes among individuals within a calendar year are common in populations with and without diabetes and that repeat hospitalizations are more frequent for some causes than others. Annual hospitalization rates including repeat hospitalizations ranged from about 10% higher for stroke to about 30% higher for heart failure in the total population, compared with those not including repeat hospitalizations. This finding suggests that hospital rates based on discharge data that include repeat hospitalizations may be considerably biased for estimating disease incidence. This is particularly true for conditions with a high proportion of repeat hospitalizations such as hyperglycemic crisis, for which inclusion of repeat hospitalizations increased rates by approximately 50%. In addition, when such data are used to examine trends, consideration should be given to the possible impact of any change in frequency of repeat hospitalizations. For example, successful efforts to reduce 30-day readmission rates could reduce repeat hospitalizations without reducing disease incidence rates among individuals.

Consistent with prior research indicating that adults with diabetes are at excess risk of hospitalization from cardiovascular disease, stroke, and lower extremity disease (including amputation) (4,13–20), our study also found that adults with diabetes were significantly more likely to be hospitalized for these conditions than adults without diabetes, even after adjusting for age. Our results also demonstrated that including repeat hospitalizations in the calculation of rates had only a modest impact on rate comparisons of populations with and without diabetes for common diabetes-related causes. For example, for acute myocardial infarction, the ratio of diabetes to non-diabetes rates was 3.9 (95% CI, 3.7–4.0) when including repeat hospitalizations versus 3.7 (95% CI, 3.5–3.8) when excluding repeats. Thus, although hospitalization rates of individuals were overestimated by including repeat hospitalizations, including them had little impact on the ratio of diabetes to non-diabetes hospitalization rates for common diabetes-related causes.

This study had several limitations. Because data from only 12 states were used to calculate hospitalization rates, our findings may not be generalizable to the national population or any one region of the country. However, our findings on the impact of repeat hospitalizations on rates and rate ratios were, for the most part, consistent across all 12 states, suggesting that the relationships described may be robust, especially because the 12 states evaluated were distributed across the country. A limitation of using administrative data, such as those from the SID, is the potential for inaccurate coding, and coding practices may vary across hospitals and states. The extent to which diabetes status is misclassified due to diabetes not being listed or incorrectly listed on discharge abstracts is unknown. Finally, because BRFSS included only noninstitutionalized people, our denominators for hospitalization rates were underestimated for both the diabetic and non-diabetic populations. Hospitalization rates are overestimated for both persons with and without diabetes because institutionalized people were not included in the denominator but were included in the numerator.

Surveillance practice commonly uses hospitalization data that include repeat hospitalizations among individuals to monitor the burden of disease. To our knowledge, ours is the first study to quantify the overestimation of hospitalization rates of individuals for common diabetes-related causes due to the inclusion of repeat hospitalizations and to determine whether this in turns affects comparisons of diabetes to non-diabetes rates. The results of this study demonstrated that the use of repeat hospitalizations substantially overestimated hospitalization rates for both the diabetic and non-diabetic populations and that this overestimation varied by cause. However, the inclusion of repeat hospitalizations for common diabetes-related causes had little impact on the comparison of rates by diabetes status, suggesting that such surveillance data may provide valid information on relative rates by diabetes status.

## Appendix Cause-Specific Hospitalization Rates With and Without Repeat Hospitalizations Among Adults With and Without Diabetes for Selected Causes, by State, 2011.

This file is available for download as a Microsoft Word document.
