# Supplementary material for: The Impact of Repeat Hospitalizations on Hospitalization Rates for Selected Conditions Among Adults With and Without Diabetes, 12 US States, 2011
Source: Prev Chronic Dis. 2015 Nov 19;12:E200. doi: 10.5888/pcd12.150274 (PMC4655479; doi:10.5888/pcd12.150274)
Supplement: Supplementary file 1 [file 15_0274_Appendix.docx]

**Appendix. Cause-Specific Hospitalization Rates With and Without Repeat Hospitalizations Among Adults With and Without Diabetes for Selected Causes, by State, 2011^a^**

| **State** | **Repeat Hospitalizations Included** | **Hospitalization Rates (per 100 Population) (95% Confidence Interval)** | | | |
| --- | --- | --- | --- | --- | --- |
|  |  | **Diabetes** | **Non–diabetes** | **Total** | **Rate Ratio (Diabetes/non–diabetes)** |
| **Acute myocardial infarction** | | | | | |
| Arkansas | Yes | 0.66 (0.59–0.73) | 0.21 (0.20–0.22) | 0.27 (0.26–0.28) | 3.16 (2.79–3.53) |
|  | No | 0.60 (0.53–0.67) | 0.20 (0.19–0.20) | 0.25 (0.24–0.26) | 3.06 (2.70–3.42) |
|  | % Increase | 9.82 | 6.19 | 7.70 | — |
| California | Yes | 0.64 (0.59–0.68) | 0.15 (0.14–0.15) | 0.21 (0.20–0.21) | 4.32 (3.99–4.65) |
|  | No | 0.53 (0.49–0.57) | 0.13 (0.13–0.13) | 0.18 (0.18–0.19) | 4.08 (3.77–4.40) |
|  | % Increase | 19.76 | 13.23 | 15.93 | — |
| Florida | Yes | 0.72 (0.65–0.79) | 0.17 (0.17–0.18) | 0.24 (0.23–0.25) | 4.12 (3.68–4.55) |
|  | No | 0.63 (0.57–0.69) | 0.16 (0.16–0.17) | 0.22 (0.21–0.22) | 3.90 (3.50–4.31) |
|  | % Increase | 14.37 | 8.44 | 10.61 | — |
| Hawaii | Yes | 0.89 (0.80–0.98) | 0.15 (0.14–0.16) | 0.23 (0.22–0.24) | 5.91 (5.25–6.58) |
|  | No | 0.79 (0.70–0.87) | 0.14 (0.13–0.14) | 0.21 (0.20–0.22) | 5.76 (5.11–6.42) |
|  | % Increase | 12.94 | 10.07 | 11.88 | — |
| Iowa | Yes | 0.77 (0.67–0.86) | 0.17 (0.17–0.18) | 0.23 (0.22–0.24) | 4.43 (3.86–5.00) |
|  | No | 0.69 (0.60–0.77) | 0.16 (0.16–0.17) | 0.21 (0.21–0.22) | 4.18 (3.65–4.71) |
|  | % Increase | 12.1 | 5.79 | 7.31 | — |
| Massachusetts | Yes | 0.70 (0.65–0.75) | 0.17 (0.17–0.18) | 0.24 (0.23–0.24) | 4.01 (3.71–4.31) |
|  | No | 0.59 (0.55–0.63) | 0.16 (0.15–0.16) | 0.21 (0.20–0.21) | 3.81 (3.52–4.10) |
|  | % Increase | 18.48 | 12.72 | 15.23 | — |
| Mississippi | Yes | 0.63 (0.58–0.69) | 0.23 (0.22–0.24) | 0.29 (0.28–0.29) | 2.77 (2.52–3.03) |
|  | No | 0.55 (0.50–0.60) | 0.20 (0.19–0.21) | 0.25 (0.24–0.26) | 2.74 (2.49–2.99) |
|  | % Increase | 14.9 | 13.52 | 14.03 | — |
| Nebraska | Yes | 0.47 (0.44–0.50) | 0.17 (0.17–0.18) | 0.21 (0.20–0.21) | 2.70 (2.53–2.87) |
|  | No | 0.43 (0.40–0.45) | 0.16 (0.16–0.17) | 0.19 (0.19–0.20) | 2.61 (2.44–2.77) |
|  | % Increase | 10.16 | 6.26 | 7.77 | — |
| New Mexico | Yes | 0.52 (0.47–0.56) | 0.14 (0.13–0.14) | 0.19 (0.18–0.19) | 3.71 (3.36–4.06) |
|  | No | 0.48 (0.44–0.52) | 0.13 (0.13–0.14) | 0.18 (0.17–0.18) | 3.56 (3.23–3.90) |
|  | % Increase | 7.36 | 3.22 | 4.70 | — |
| New York | Yes | 0.55 (0.50–0.60) | 0.16 (0.15–0.16) | 0.22 (0.21–0.22) | 3.47 (3.13–3.81) |
|  | No | 0.47 (0.43–0.51) | 0.14 (0.14–0.15) | 0.19 (0.19–0.20) | 3.28 (2.96–3.60) |
|  | % Increase | 16.75 | 10.32 | 12.90 | — |
| Vermont | Yes | 0.61 (0.54–0.68) | 0.13 (0.12–0.13) | 0.18 (0.17–0.18) | 4.69 (4.11–5.26) |
|  | No | 0.54 (0.47–0.61) | 0.12 (0.12–0.13) | 0.17 (0.16–0.17) | 4.33 (3.76–4.91) |
|  | % Increase | 12.92 | 4.4 | 7.11 | — |
| Washington | Yes | 0.48 (0.44–0.52) | 0.15 (0.14–0.15) | 0.19 (0.19–0.20) | 3.21 (2.95–3.48) |
|  | No | 0.43 (0.39–0.46) | 0.14 (0.14–0.14) | 0.18 (0.17–0.18) | 3.07 (2.82–3.32) |
|  | % Increase | 12.01 | 7 | 8.68 | — |
| **Heart failure** | | | | | |
| Arkansas | Yes | 1.31 (1.17–1.44) | 0.27 (0.26–0.29) | 0.44 (0.42–0.46) | 4.77 (4.22–5.32) |
|  | No | 0.92 (0.83–1.01) | 0.22 (0.21–0.23) | 0.33 (0.31–0.34) | 4.23 (3.74–4.72) |
|  | % Increase | 41.87 | 25.81 | 33.13 | — |
| California | Yes | 1.16 (1.07–1.24) | 0.19 (0.18–0.19) | 0.31 (0.30–0.32) | 6.22 (5.71–6.74) |
|  | No | 0.80 (0.74–0.85) | 0.15 (0.14–0.15) | 0.24 (0.23–0.24) | 5.35 (4.92–5.78) |
|  | % Increase | 45.33 | 24.97 | 31.56 | — |
| Florida | Yes | 1.31 (1.19–1.44) | 0.21 (0.21–0.22) | 0.36 (0.35–0.37) | 6.15 (5.52–6.78) |
|  | No | 0.92 (0.83–1.00) | 0.17 (0.16–0.18) | 0.27 (0.26–0.28) | 5.42 (4.88–5.96) |
|  | % Increase | 43.6 | 26.5 | 33.19 | — |
| Hawaii | Yes | 1.24 (1.11–1.37) | 0.16 (0.15–0.17) | 0.28 (0.27–0.29) | 7.86 (6.97–8.76) |
|  | No | 0.89 (0.80–0.98) | 0.12 (0.12–0.13) | 0.21 (0.20–0.22) | 7.16 (6.34–7.98) |
|  | % Increase | 40.11 | 27.58 | 32.48 | — |
| Iowa | Yes | 1.04 (0.94–1.15) | 0.20 (0.19–0.21) | 0.30 (0.29–0.32) | 5.19 (4.60–5.78) |
|  | No | 0.84 (0.75–0.92) | 0.17 (0.16–0.18) | 0.25 (0.24–0.26) | 4.99 (4.41–5.57) |
|  | % Increase | 24.93 | 20.04 | 22.87 | — |
| Massachusetts | Yes | 1.32 (1.22–1.42) | 0.26 (0.25–0.27) | 0.40 (0.39–0.42) | 5.06 (4.63–5.49) |
|  | No | 0.93 (0.86–0.99) | 0.21 (0.20–0.22) | 0.31 (0.29–0.32) | 4.47 (4.08–4.85) |
|  | % Increase | 42.58 | 25.86 | 31.79 | — |
| Mississippi | Yes | 1.36 (1.25–1.47) | 0.32 (0.30–0.33) | 0.49 (0.47–0.50) | 4.28 (3.88–4.68) |
|  | No | 0.97 (0.89–1.05) | 0.25 (0.23–0.26) | 0.36 (0.35–0.38) | 3.97 (3.60–4.35) |
|  | % Increase | 39.72 | 29.71 | 34.02 | — |
| Nebraska | Yes | 0.79 (0.74–0.83) | 0.21 (0.20–0.21) | 0.28 (0.27–0.29) | 3.78 (3.53–4.04) |
|  | No | 0.61 (0.57–0.64) | 0.18 (0.17–0.18) | 0.23 (0.23–0.24) | 3.46 (3.23–3.68) |
|  | % Increase | 29.99 | 18.75 | 21.40 | — |
| New Mexico | Yes | 0.64 (0.58–0.69) | 0.13 (0.12–0.14) | 0.20 (0.19–0.21) | 4.90 (4.41–5.38) |
|  | No | 0.51 (0.47–0.56) | 0.11 (0.11–0.12) | 0.17 (0.16–0.18) | 4.47 (4.04–4.90) |
|  | % Increase | 24.15 | 13.33 | 18.0 | — |
| New York | Yes | 1.08 (0.98–1.18) | 0.23 (0.22–0.24) | 0.37 (0.35–0.39) | 4.64 (4.15–5.13) |
|  | No | 0.77 (0.69–0.84) | 0.18 (0.17–0.19) | 0.28 (0.27–0.29) | 4.15 (3.71–4.60) |
|  | % Increase | 41.18 | 26.3 | 32.68 | — |
| Vermont | Yes | 0.70 (0.62–0.77) | 0.14 (0.13–0.15) | 0.21 (0.20–0.22) | 4.95 (4.36–5.55) |
|  | No | 0.58 (0.52–0.64) | 0.12 (0.11–0.12) | 0.18 (0.17–0.18) | 4.96 (4.35–5.56) |
|  | % Increase | 20.15 | 20.23 | 20.93 | — |
| Washington | Yes | 0.77 (0.71–0.84) | 0.17 (0.16–0.18) | 0.26 (0.25–0.27) | 4.51 (4.11–4.92) |
|  | No | 0.58 (0.54–0.63) | 0.14 (0.14–0.15) | 0.21 (0.20–0.21) | 4.12 (3.75–4.49) |
|  | % Increase | 32.5 | 20.9 | 25.63 | — |
| **Cardiovascular disease** | | | | | |
| Arkansas | Yes | 5.60 (5.00–6.20) | 1.72 (1.64–1.79) | 2.27 (2.19–2.36) | 3.26 (2.88–3.64) |
|  | No | 4.05 (3.61–4.48) | 1.40 (1.34–1.46) | 1.77 (1.71–1.84) | 2.90 (2.56–3.23) |
|  | % Increase | 38.32 | 22.87 | 28.16 | — |
| California | Yes | 4.62 (4.28–4.96) | 1.14 (1.11–1.17) | 1.57 (1.53–1.61) | 4.06 (3.74–4.38) |
|  | No | 3.26 (3.03–3.50) | 0.93 (0.90–0.96) | 1.22 (1.18–1.25) | 3.51 (3.24–3.79) |
|  | % Increase | 41.63 | 22.64 | 28.72 | — |
| Florida | Yes | 5.75 (5.15–6.35) | 1.43 (1.39–1.48) | 1.95 (1.90–2.00) | 4.01 (3.58–4.45) |
|  | No | 4.08 (3.67–4.48) | 1.18 (1.14–1.21) | 1.52 (1.48–1.56) | 3.47 (3.11–3.83) |
|  | % Increase | 41.1 | 21.95 | 27.94 | — |
| Hawaii | Yes | 4.95 (4.44–5.46) | 0.96 (0.92–1.00) | 1.41 (1.36–1.47) | 5.15 (4.57–5.72) |
|  | No | 3.67 (3.29–4.05) | 0.80 (0.77–0.84) | 1.12 (1.08–1.16) | 4.56 (4.05–5.07) |
|  | % Increase | 35.14 | 19.81 | 26.03 | — |
| Iowa | Yes | 4.67 (4.16–5.18) | 1.24 (1.19–1.28) | 1.59 (1.54–1.65) | 3.78 (3.35–4.22) |
|  | No | 3.74 (3.32–4.17) | 1.05 (1.01–1.08) | 1.32 (1.27–1.36) | 3.58 (3.16–4.00) |
|  | % Increase | 24.8 | 18.11 | 21.15 | — |
| Massachusetts | Yes | 5.01 (4.67–5.36) | 1.45 (1.40–1.49) | 1.88 (1.83–1.93) | 3.47 (3.21–3.73) |
|  | No | 3.54 (3.30–3.78) | 1.17 (1.13–1.20) | 1.45 (1.41–1.49) | 3.04 (2.81–3.27) |
|  | % Increase | 41.64 | 24.04 | 29.38 | — |
| Mississippi | Yes | 5.27 (4.83–5.72) | 1.74 (1.68–1.80) | 2.27 (2.20–2.33) | 3.02 (2.75–3.30) |
|  | No | 3.77 (3.45–4.09) | 1.36 (1.32–1.41) | 1.71 (1.66–1.76) | 2.77 (2.51–3.02) |
|  | % Increase | 39.79 | 27.87 | 32.25 | — |
| Nebraska | Yes | 3.71 (3.49–3.93) | 1.35 (1.32–1.38) | 1.62 (1.59–1.66) | 2.74 (2.57–2.91) |
|  | No | 2.85 (2.68–3.02) | 1.15 (1.12–1.18) | 1.34 (1.32–1.37) | 2.48 (2.33–2.64) |
|  | % Increase | 30.04 | 17.74 | 20.92 | — |
| New Mexico | Yes | 3.03 (2.76–3.30) | 0.88 (0.85–0.91) | 1.16 (1.13–1.20) | 3.43 (3.10–3.76) |
|  | No | 2.47 (2.25–2.69) | 0.78 (0.75–0.80) | 0.99 (0.96–1.02) | 3.18 (2.88–3.49) |
|  | % Increase | 22.82 | 13.93 | 17.19 | — |
| New York | Yes | 4.70 (4.27–5.13) | 1.44 (1.39–1.50) | 1.92 (1.85–1.99) | 3.26 (2.93–3.58) |
|  | No | 3.40 (3.09–3.71) | 1.18 (1.13–1.23) | 1.50 (1.45–1.55) | 2.88 (2.59–3.16) |
|  | % Increase | 38.44 | 22.37 | 28.1 | — |
| Vermont | Yes | 3.10 (2.79–3.41) | 0.89 (0.86–0.93) | 1.14 (1.10–1.18) | 3.47 (3.09–3.85) |
|  | No | 2.55 (2.29–2.82) | 0.78 (0.75–0.81) | 0.97 (0.94–1.01) | 3.28 (2.91–3.65) |
|  | % Increase | 21.31 | 14.57 | 17.21 | — |
| Washington | Yes | 3.28 (3.03–3.53) | 1.09 (1.06–1.13) | 1.38 (1.34–1.42) | 3.01 (2.76–3.26) |
|  | No | 2.49 (2.30–2.68) | 0.92 (0.89–0.95) | 1.12 (1.09–1.16) | 2.71 (2.49–2.94) |
|  | % Increase | 31.85 | 18.75 | 22.83 | — |
| **Ischemic heart disease** | | | | | |
| Arkansas | Yes | 1.87 (1.66–2.07) | 0.54 (0.52–0.56) | 0.73 (0.70–0.75) | 3.45 (3.05–3.85) |
|  | No | 1.56 (1.39–1.72) | 0.48 (0.46–0.50) | 0.63 (0.61–0.66) | 3.23 (2.86–3.59) |
|  | % Increase | 19.8 | 11.97 | 14.64 | — |
| California | Yes | 1.33 (1.24–1.43) | 0.29 (0.28–0.29) | 0.42 (0.41–0.43) | 4.67 (4.32–5.02) |
|  | No | 1.07 (0.99–1.14) | 0.24 (0.24–0.25) | 0.35 (0.34–0.36) | 4.35 (4.03–4.68) |
|  | % Increase | 24.95 | 16.51 | 19.77 | — |
| Florida | Yes | 1.64 (1.47–1.80) | 0.36 (0.35–0.37) | 0.51 (0.50–0.53) | 4.58 (4.10–5.05) |
|  | No | 1.35 (1.22–1.48) | 0.32 (0.31–0.33) | 0.44 (0.43–0.46) | 4.24 (3.81–4.68) |
|  | % Increase | 21.18 | 12.41 | 15.23 | — |
| Hawaii | Yes | 1.54 (1.39–1.70) | 0.25 (0.24–0.26) | 0.40 (0.38–0.41) | 6.21 (5.53–6.89) |
|  | No | 1.29 (1.15–1.42) | 0.22 (0.21–0.23) | 0.34 (0.33–0.35) | 5.90 (5.25–6.54) |
|  | % Increase | 20.04 | 14.05 | 16.81 | — |
| Iowa | Yes | 1.56 (1.38–1.74) | 0.34 (0.32–0.35) | 0.46 (0.44–0.47) | 4.65 (4.10–5.21) |
|  | No | 1.31 (1.16–1.46) | 0.30 (0.29–0.31) | 0.40 (0.39–0.41) | 4.36 (3.85–4.88) |
|  | % Increase | 18.9 | 11.45 | 13.39 | — |
| Massachusetts | Yes | 1.37 (1.28–1.46) | 0.30 (0.30–0.31) | 0.43 (0.42–0.44) | 4.50 (4.17–4.83) |
|  | No | 1.09 (1.02–1.17) | 0.26 (0.25–0.27) | 0.35 (0.35–0.36) | 4.22 (3.91–4.53) |
|  | % Increase | 25.58 | 17.79 | 20.88 | — |
| Mississippi | Yes | 1.39 (1.28–1.51) | 0.45 (0.44–0.47) | 0.59 (0.58–0.61) | 3.09 (2.82–3.36) |
|  | No | 1.15 (1.06–1.25) | 0.38 (0.37–0.40) | 0.50 (0.49–0.51) | 2.99 (2.73–3.26) |
|  | % Increase | 20.87 | 17.21 | 18.41 | — |
| Nebraska | Yes | 1.10 (1.03–1.16) | 0.35 (0.34–0.35) | 0.43 (0.42–0.44) | 3.18 (2.98–3.37) |
|  | No | 0.95 (0.90–1.01) | 0.31 (0.31–0.32) | 0.39 (0.38–0.39) | 3.04 (2.85–3.24) |
|  | % Increase | 14.93 | 10.16 | 11.83 | — |
| New Mexico | Yes | 0.93 (0.86–1.01) | 0.24 (0.23–0.25) | 0.33 (0.32–0.34) | 3.87 (3.53–4.21) |
|  | No | 0.83 (0.76–0.90) | 0.22 (0.22–0.23) | 0.30 (0.29–0.31) | 3.71 (3.38–4.03) |
|  | % Increase | 12.74 | 7.99 | 9.78 | — |
| New York | Yes | 1.50 (1.36–1.63) | 0.38 (0.36–0.39) | 0.54 (0.52–0.55) | 3.98 (3.60–4.37) |
|  | No | 1.21 (1.10–1.32) | 0.33 (0.31–0.34) | 0.45 (0.44–0.47) | 3.72 (3.36–4.08) |
|  | % Increase | 23.36 | 15.27 | 18.54 | — |
| Vermont | Yes | 1.02 (0.91–1.14) | 0.21 (0.20–0.21) | 0.29 (0.28–0.30) | 4.94 (4.37–5.51) |
|  | No | 0.88 (0.79–0.98) | 0.19 (0.19–0.20) | 0.26 (0.26–0.27) | 4.54 (4.02–5.06) |
|  | % Increase | 15.91 | 6.51 | 9.13 | — |
| Washington | Yes | 0.89 (0.83–0.96) | 0.26 (0.25–0.26) | 0.34 (0.33–0.35) | 3.48 (3.20–3.76) |
|  | No | 0.75 (0.70–0.81) | 0.23 (0.22–0.24) | 0.30 (0.29–0.30) | 3.29 (3.03–3.55) |
|  | % Increase | 18.33 | 11.82 | 13.95 | — |
| **Stroke** | | | | | |
| Arkansas | Yes | 0.72 (0.65–0.79) | 0.27 (0.26–0.28) | 0.34 (0.32–0.35) | 2.68 (2.38–2.97) |
|  | No | 0.64 (0.58–0.70) | 0.24 (0.23–0.25) | 0.30 (0.29–0.31) | 2.64 (2.35–2.93) |
|  | % Increase | 12.78 | 11.28 | 11.73 | — |
| California | Yes | 0.69 (0.64–0.73) | 0.21 (0.21–0.22) | 0.27 (0.27–0.28) | 3.20 (2.95–3.44) |
|  | No | 0.60 (0.56–0.64) | 0.19 (0.19–0.20) | 0.25 (0.24–0.25) | 3.08 (2.85–3.32) |
|  | % Increase | 14.1 | 10.08 | 11.3 | — |
| Florida | Yes | 0.74 (0.68–0.81) | 0.23 (0.22–0.24) | 0.29 (0.29–0.30) | 3.26 (2.95–3.57) |
|  | No | 0.65 (0.59–0.71) | 0.21 (0.20–0.22) | 0.27 (0.26–0.27) | 3.12 (2.83–3.41) |
|  | % Increase | 14.03 | 9.3 | 10.7 | — |
| Hawaii | Yes | 0.77 (0.69–0.85) | 0.20 (0.19–0.21) | 0.27 (0.26–0.28) | 3.78 (3.35–4.21) |
|  | No | 0.68 (0.61–0.76) | 0.18 (0.17–0.20) | 0.24 (0.23–0.25) | 3.70 (3.24–4.16) |
|  | % Increase | 13.15 | 10.69 | 11.16 | — |
| Iowa | Yes | 0.63 (0.57–0.69) | 0.21 (0.20–0.21) | 0.25 (0.24–0.26) | 3.07 (2.75–3.40) |
|  | No | 0.58 (0.52–0.64) | 0.19 (0.18–0.20) | 0.23 (0.22–0.24) | 3.08 (2.74–3.42) |
|  | % Increase | 8.63 | 9.05 | 9.42 | — |
| Massachusetts | Yes | 0.58 (0.55–0.62) | 0.24 (0.23–0.24) | 0.28 (0.27–0.29) | 2.49 (2.30–2.67) |
|  | No | 0.53 (0.49–0.56) | 0.22 (0.21–0.22) | 0.26 (0.25–0.26) | 2.45 (2.27–2.64) |
|  | % Increase | 10.41 | 9.01 | 9.46 | — |
| Mississippi | Yes | 0.79 (0.73–0.86) | 0.31 (0.30–0.32) | 0.39 (0.37–0.40) | 2.56 (2.34–2.78) |
|  | No | 0.68 (0.63–0.74) | 0.27 (0.26–0.28) | 0.33 (0.32–0.35) | 2.51 (2.29–2.73) |
|  | % Increase | 16.51 | 14.44 | 15.45 | — |
| Nebraska | Yes | 0.52 (0.49–0.55) | 0.22 (0.22–0.23) | 0.26 (0.25–0.26) | 2.36 (2.21–2.51) |
|  | No | 0.47 (0.44–0.49) | 0.20 (0.20–0.21) | 0.23 (0.23–0.24) | 2.29 (2.14–2.43) |
|  | % Increase | 12.35 | 8.97 | 9.79 | — |
| New Mexico | Yes | 0.45 (0.41–0.49) | 0.15 (0.15–0.16) | 0.19 (0.19–0.20) | 2.92 (2.64–3.19) |
|  | No | 0.41 (0.37–0.44) | 0.14 (0.14–0.15) | 0.18 (0.17–0.18) | 2.86 (2.60–3.13) |
|  | % Increase | 9.56 | 7.59 | 8.29 | — |
| New York | Yes | 0.56 (0.51–0.61) | 0.21 (0.21–0.22) | 0.27 (0.26–0.28) | 2.61 (2.36–2.87) |
|  | No | 0.50 (0.46–0.55) | 0.20 (0.19–0.21) | 0.24 (0.23–0.25) | 2.53 (2.28–2.78) |
|  | % Increase | 11.46 | 8.03 | 9.12 | — |
| Vermont | Yes | 0.44 (0.39–0.49) | 0.16 (0.15–0.17) | 0.19 (0.18–0.20) | 2.74 (2.42–3.05) |
|  | No | 0.40 (0.33–0.47) | 0.15 (0.12–0.17) | 0.17 (0.15–0.20) | 2.72 (2.07–3.37) |
|  | % Increase | 11.05 | 10.49 | 10.78 | —— |
| Washington | Yes | 0.52 (0.48–0.56) | 0.22 (0.21–0.23) | 0.26 (0.25–0.27) | 2.38 (2.18–2.58) |
|  | No | 0.47 (0.43–0.50) | 0.20 (0.19–0.21) | 0.24 (0.23–0.24) | 2.33 (2.13–2.52) |
|  | % Increase | 11.53 | 8.95 | 9.73 | — |
| **Lower extremity amputation** | | | | | |
| Arkansas | Yes | 0.35 (0.29–0.40) | 0.02 (0.02–0.02) | 0.06 (0.06–0.06) | 19.16 (16.22–22.10) |
|  | No | 0.29 (0.25–0.33) | 0.02 (0.02–0.02) | 0.05 (0.05–0.05) | 17.66 (15.01–20.30) |
|  | % Increase | 18.5 | 9.2 | 14.98 | — |
| California | Yes | 0.36 (0.33–0.40) | 0.01 (0.01–0.01) | 0.04 (0.04–0.05) | 35.56 (31.83–39.28) |
|  | No | 0.31 (0.28–0.34) | 0.01 (0.01–0.01) | 0.04 (0.04–0.04) | 32.84 (29.39–36.29) |
|  | % Increase | 18.24 | 9.2 | 16.82 | — |
| Florida | Yes | 0.36 (0.31–0.42) | 0.01 (0.01–0.01) | 0.05 (0.05–0.05) | 30.48 (25.94–35.01) |
|  | No | 0.31 (0.26–0.35) | 0.01 (0.01–0.01) | 0.04 (0.04–0.04) | 28.22 (23.98–32.45) |
|  | % Increase | 17.57 | 8.86 | 16.14 | — |
| Hawaii | Yes | 0.44 (0.38–0.50) | 0.01 (0.01–0.01) | 0.05 (0.05–0.05) | 44.82 (38.46–51.18) |
|  | No | 0.39 (0.34–0.45) | 0.01 (0.01–0.01) | 0.04 (0.04–0.04) | 43.87 (37.39–50.35) |
|  | % Increase | 11.69 | 9.34 | 14.76 | — |
| Iowa | Yes | 0.30 (0.25–0.35) | 0.01 (0.01–0.01) | 0.03 (0.03–0.03) | 33.69 (27.93–39.45) |
|  | No | 0.27 (0.22–0.31) | 0.01 (0.01–0.01) | 0.03 (0.03–0.03) | 32.19 (26.42–37.97) |
|  | % Increase | 14.19 | 9.11 | 12.08 | — |
| Massachusetts | Yes | 0.32 (0.30–0.35) | 0.01 (0.01–0.01) | 0.04 (0.04–0.05) | 25.78 (23.59–27.98) |
|  | No | 0.27 (0.25–0.29) | 0.01 (0.01–0.01) | 0.04 (0.04–0.04) | 23.71 (21.67–25.76) |
|  | % Increase | 20.62 | 10.94 | 18.40 | — |
| Mississippi | Yes | 0.41 (0.36–0.46) | 0.02 (0.02–0.02) | 0.07 (0.07–0.07) | 17.60 (15.41–19.78) |
|  | No | 0.34 (0.30–0.38) | 0.02 (0.02–0.02) | 0.06 (0.06–0.06) | 16.47 (14.40–18.54) |
|  | % Increase | 21.37 | 13.59 | 18.62 | — |
| Nebraska | Yes | 0.40 (0.36–0.44) | 0.02 (0.02–0.02) | 0.05 (0.05–0.05) | 23.96 (21.61–26.31) |
|  | No | 0.35 (0.32–0.38) | 0.02 (0.02–0.02) | 0.04 (0.04–0.04) | 22.54 (20.33–24.74) |
|  | % Increase | 14.03 | 7.26 | 12.1 | — |
| New Mexico | Yes | 0.29 (0.25–0.33) | 0.01 (0.01–0.01) | 0.04 (0.04–0.04) | 29.74 (25.66–33.82) |
|  | No | 0.26 (0.22–0.29) | 0.01 (0.01–0.01) | 0.03 (0.03–0.04) | 27.21 (23.39–31.02) |
|  | % Increase | 12.53 | 2.93 | 11.87 | — |
| New York | Yes | 0.33 (0.29–0.37) | 0.01 (0.01–0.01) | 0.05 (0.05–0.05) | 28.52 (24.83–32.20) |
|  | No | 0.28 (0.25–0.32) | 0.01 (0.01–0.01) | 0.04 (0.04–0.04) | 26.27 (22.86–29.69) |
|  | % Increase | 18.56 | 9.23 | 17.31 | — |
| Vermont | Yes | 0.26 (0.21–0.32) | 0.01 (0.01–0.01) | 0.02 (0.02–0.02) | 43.48 (34.52–52.43) |
|  | No | 0.21 (0.17–0.25) | 0.01 (0.01–0.01) | 0.02 (0.02–0.02) | 37.95 (30.64–45.26) |
|  | % Increase | 23.8 | 8.06 | 14.39 | — |
| Washington | Yes | 0.26 (0.23–0.29) | 0.01 (0.01–0.01) | 0.04 (0.04–0.04) | 22.41 (19.89–24.92) |
|  | No | 0.22 (0.20–0.24) | 0.01 (0.01–0.01) | 0.03 (0.03–0.03) | 20.28 (18.05–22.51) |
|  | % Increase | 18.72 | 7.46 | 14.48 | — |
| **Peripheral arterial disease** | | | | | |
| Arkansas | Yes | 0.32 (0.29–0.36) | 0.06 (0.06–0.06) | 0.10 (0.10–0.10) | 5.41 (4.83–6.00) |
|  | No | 0.27 (0.24–0.29) | 0.05 (0.05–0.05) | 0.08 (0.08–0.09) | 5.32 (4.74–5.90) |
|  | % Increase | 21.18 | 19.12 | 20.77 | — |
| California | Yes | 0.31 (0.29–0.33) | 0.03 (0.03–0.03) | 0.06 (0.06–0.07) | 11.06 (10.19–11.92) |
|  | No | 0.24 (0.23–0.26) | 0.02 (0.02–0.02) | 0.05 (0.05–0.05) | 10.15 (9.34–10.95) |
|  | % Increase | 25.59 | 15.25 | 22.74 | — |
| Florida | Yes | 0.31 (0.28–0.34) | 0.04 (0.04–0.04) | 0.08 (0.07–0.08) | 7.98 (7.21–8.75) |
|  | No | 0.25 (0.23–0.27) | 0.03 (0.03–0.04) | 0.06 (0.06–0.06) | 7.38 (6.67–8.10) |
|  | % Increase | 23.41 | 14.13 | 19.2 | — |
| Hawaii | Yes | 0.30 (0.27–0.34) | 0.02 (0.02–0.02) | 0.05 (0.05–0.05) | 16.36 (14.48–18.23) |
|  | No | 0.25 (0.23–0.28) | 0.02 (0.02–0.02) | 0.04 (0.04–0.04) | 15.78 (13.90–17.65) |
|  | % Increase | 20.06 | 15.78 | 20.27 | — |
| Iowa | Yes | 0.21 (0.19–0.24) | 0.03 (0.03–0.03) | 0.05 (0.05–0.05) | 6.67 (5.94–7.40) |
|  | No | 0.19 (0.17–0.21) | 0.03 (0.03–0.03) | 0.05 (0.04–0.05) | 6.64 (5.82–7.46) |
|  | % Increase | 12.35 | 11.81 | 13.43 | — |
| Massachusetts | Yes | 0.36 (0.34–0.38) | 0.05 (0.04–0.05) | 0.09 (0.08–0.09) | 7.83 (7.25–8.40) |
|  | No | 0.28 (0.27–0.30) | 0.04 (0.04–0.04) | 0.07 (0.07–0.07) | 7.23 (6.70–7.76) |
|  | % Increase | 26.06 | 16.51 | 21.49 | — |
| Mississippi | Yes | 0.40 (0.36–0.43) | 0.06 (0.06–0.06) | 0.11 (0.11–0.11) | 6.50 (5.88–7.13) |
|  | No | 0.30 (0.27–0.33) | 0.05 (0.05–0.05) | 0.09 (0.09–0.09) | 5.94 (5.37–6.51) |
|  | % Increase | 32.06 | 20.65 | 25.76 | — |
| Nebraska | Yes | 0.24 (0.22–0.25) | 0.04 (0.04–0.04) | 0.07 (0.07–0.07) | 5.69 (5.34–6.03) |
|  | No | 0.20 (0.19–0.22) | 0.04 (0.04–0.04) | 0.06 (0.06–0.06) | 5.54 (5.20–5.88) |
|  | % Increase | 16 | 13.06 | 15.63 | — |
| New Mexico | Yes | 0.19 (0.17–0.21) | 0.02 (0.02–0.02) | 0.05 (0.04–0.05) | 8.42 (7.66–9.18) |
|  | No | 0.16 (0.15–0.18) | 0.02 (0.02–0.02) | 0.04 (0.04–0.04) | 7.91 (7.18–8.63) |
|  | % Increase | 15.65 | 8.6 | 13.54 | — |
| New York | Yes | 0.38 (0.34–0.41) | 0.04 (0.04–0.04) | 0.09 (0.09–0.10) | 8.91 (8.01–9.81) |
|  | No | 0.30 (0.27–0.32) | 0.04 (0.03–0.04) | 0.07 (0.07–0.08) | 8.15 (7.31–8.98) |
|  | % Increase | 27.67 | 16.72 | 24.96 | — |
| Vermont | Yes | 0.21 (0.18–0.24) | 0.02 (0.02–0.02) | 0.03 (0.03–0.04) | 12.21 (10.36–14.05) |
|  | No | 0.19 (0.16–0.22) | 0.02 (0.02–0.02) | 0.03 (0.03–0.03) | 11.54 (9.62–13.47) |
|  | % Increase | 11.22 | 5.19 | 10.74 | — |
| Washington | Yes | 0.20 (0.18–0.21) | 0.03 (0.03–0.03) | 0.06 (0.05–0.06) | 5.86 (5.38–6.33) |
|  | No | 0.16 (0.15–0.17) | 0.03 (0.03–0.03) | 0.05 (0.05–0.05) | 5.43 (4.99–5.86) |
|  | % Increase | 21.12 | 12.23 | 16.29 | — |
| **Lower extremity ulcer** | | | | | |
| Arkansas | Yes | 0.58 (0.48–0.69) | 0.11 (0.10–0.11) | 0.16 (0.16–0.17) | 5.37 (4.41–6.33) |
|  | No | 0.50 (0.41–0.59) | 0.10 (0.09–0.10) | 0.14 (0.14–0.14) | 5.21 (4.27–6.16) |
|  | % Increase | 16.98 | 13.57 | 15.40 | — |
| California | Yes | 0.59 (0.52–0.65) | 0.09 (0.09–0.10) | 0.14 (0.13–0.14) | 6.20 (5.49–6.91) |
|  | No | 0.50 (0.44–0.55) | 0.08 (0.08–0.08) | 0.12 (0.11–0.12) | 5.99 (5.30–6.68) |
|  | % Increase | 18.02 | 14.08 | 15.49 | — |
| Florida | Yes | 0.72 (0.60–0.85) | 0.13 (0.13–0.13) | 0.18 (0.18–0.19) | 5.57 (4.62–6.52) |
|  | No | 0.63 (0.52–0.73) | 0.12 (0.11–0.12) | 0.16 (0.16–0.16) | 5.39 (4.47–6.30) |
|  | % Increase | 15.47 | 11.68 | 12.67 | — |
| Hawaii | Yes | 0.90 (0.76–1.04) | 0.13 (0.12–0.13) | 0.18 (0.18–0.19) | 7.18 (6.03–8.33) |
|  | No | 0.78 (0.65–0.90) | 0.11 (0.11–0.11) | 0.16 (0.16–0.16) | 7.12 (5.97–8.27) |
|  | % Increase | 15.75 | 14.87 | 15.35 | — |
| Iowa | Yes | 1.12 (0.89–1.34) | 0.11 (0.11–0.11) | 0.17 (0.16–0.17) | 10.30 (8.16–12.43) |
|  | No | 0.89 (0.71–1.08) | 0.09 (0.09–0.10) | 0.14 (0.14–0.14) | 9.56 (7.57–11.55) |
|  | % Increase | 24.83 | 15.91 | 19.31 | — |
| Massachusetts | Yes | 0.68 (0.61–0.74) | 0.14 (0.14–0.14) | 0.19 (0.18–0.19) | 4.80 (4.34–5.27) |
|  | No | 0.56 (0.51–0.61) | 0.12 (0.12–0.13) | 0.16 (0.16–0.17) | 4.56 (4.13–4.98) |
|  | % Increase | 21.03 | 14.79 | 15.67 | — |
| Mississippi | Yes | 0.62 (0.53–0.70) | 0.13 (0.13–0.14) | 0.18 (0.18–0.19) | 4.69 (4.04–5.34) |
|  | No | 0.53 (0.46–0.60) | 0.12 (0.11–0.12) | 0.16 (0.16–0.16) | 4.55 (3.91–5.18) |
|  | % Increase | 16.33 | 12.7 | 14.16 | — |
| Nebraska | Yes | 0.62 (0.56–0.68) | 0.10 (0.10–0.10) | 0.14 (0.14–0.14) | 6.28 (5.68–6.89) |
|  | No | 0.54 (0.49–0.59) | 0.09 (0.09–0.09) | 0.13 (0.12–0.13) | 5.99 (5.41–6.57) |
|  | % Increase | 14.81 | 9.5 | 11.55 | — |
| New Mexico | Yes | 0.49 (0.42–0.57) | 0.09 (0.09–0.10) | 0.13 (0.12–0.13) | 5.27 (4.43–6.12) |
|  | No | 0.44 (0.37–0.51) | 0.09 (0.08–0.09) | 0.12 (0.11–0.12) | 5.12 (4.30–5.94) |
|  | % Increase | 11.38 | 8.16 | 9.08 | — |
| New York | Yes | 0.59 (0.51–0.66) | 0.14 (0.13–0.14) | 0.19 (0.18–0.19) | 4.27 (3.72–4.83) |
|  | No | 0.50 (0.44–0.56) | 0.12 (0.12–0.13) | 0.16 (0.16–0.17) | 4.12 (3.60–4.65) |
|  | % Increase | 17.22 | 13.13 | 14.31 | — |
| Vermont | Yes | 0.54 (0.44–0.64) | 0.08 (0.08–0.08) | 0.11 (0.11–0.12) | 6.87 (5.60–8.13) |
|  | No | 0.48 (0.39–0.57) | 0.07 (0.07–0.07) | 0.10 (0.10–0.11) | 6.69 (5.38–8.00) |
|  | % Increase | 12.39 | 9.52 | 12.02 | — |
| Washington | Yes | 0.47 (0.41–0.53) | 0.09 (0.09–0.09) | 0.12 (0.12–0.12) | 5.28 (4.58–5.98) |
|  | No | 0.40 (0.35–0.45) | 0.08 (0.08–0.08) | 0.11 (0.10–0.11) | 5.08 (4.42–5.75) |
|  | % Increase | 18.44 | 13.97 | 14.93 | — |
| **Hypoglycemia** | | | | | |
| Arkansas | Yes | 0.25 (0.21–0.29) | — | | |
|  | No | 0.23 (0.20–0.27) |  |  |  |
|  | % Increase | 7.72 |  |  |  |
| California | Yes | 0.31 (0.28–0.34) | — | | |
|  | No | 0.28 (0.25–0.30) |  |  |  |
|  | % Increase | 10.87 |  |  |  |
| Florida | Yes | 0.34 (0.29–0.40) | — | | |
|  | No | 0.31 (0.26–0.36) |  |  |  |
|  | % Increase | 11.15 |  |  |  |
| Hawaii | Yes | 0.26 (0.22–0.29) | — | | |
|  | No | 0.24 (0.21–0.27) |  |  |  |
|  | % Increase | 5.8 |  |  |  |
| Iowa | Yes | 0.31 (0.24–0.38) | — | | |
|  | No | 0.29 (0.23–0.35) |  |  |  |
|  | % Increase | 8.72 |  |  |  |
| Massachusetts | Yes | 0.32 (0.29–0.35) | — | | |
|  | No | 0.30 (0.27–0.33) |  |  |  |
|  | % Increase | 7.13 |  |  |  |
| Mississippi | Yes | 0.31 (0.28–0.35) | — | | |
|  | No | 0.28 (0.25–0.31) |  |  |  |
|  | % Increase | 10.56 |  |  |  |
| Nebraska | Yes | 0.19 (0.17–0.21) | — | | |
|  | No | 0.18 (0.17–0.20) |  |  |  |
|  | % Increase | 4.01 |  |  |  |
| New Mexico | Yes | 0.21 (0.18–0.24) | — | | |
|  | No | 0.19 (0.17–0.22) |  |  |  |
|  | Increase | 7.77 |  |  |  |
| New York | Yes | 0.35 (0.31–0.40) | — | | |
|  | No | 0.32 (0.29–0.36) |  |  |  |
|  | % Increase | 9.46 |  |  |  |
| Vermont | Yes | 0.12 (0.10–0.14) | — | | |
|  | No | 0.12 (0.10–0.14) |  |  |  |
|  | % Increase | 0 |  |  |  |
| Washington | Yes | 0.18 (0.15–0.20) | — | | |
|  | No | 0.16 (0.14–0.18) |  |  |  |
|  | % Increase | 11.34 |  |  |  |
| **Hyperglycemic crisis** | | | | | |
| Arkansas | Yes | 1.89 (1.25–2.52) | — | | |
|  | No | 1.12 (0.76–1.48) |  |  |  |
|  | % Increase | 68.6 |  |  |  |
| California | Yes | 1.72 (1.38–2.06) | — | | |
|  | No | 1.14 (0.92–1.36) |  |  |  |
|  | % Increase | 50.24 |  |  |  |
| Florida | Yes | 2.01 (1.41–2.60) | — | | |
|  | No | 1.32 (0.94–1.71) |  |  |  |
|  | % Increase | 51.51 |  |  |  |
| Hawaii | Yes | 1.02 (0.76–1.27) | — | | |
|  | No | 0.64 (0.49–0.80) |  |  |  |
|  | % Increase | 57.58 |  |  |  |
| Iowa | Yes | 3.23 (2.23–4.23) | — | | |
|  | No | 2.03 (1.41–2.66) |  |  |  |
|  | % Increase | 59.04 |  |  |  |
| Massachusetts | Yes | 1.40 (1.12–1.68) | — | | |
|  | No | 0.92 (0.75–1.10) |  |  |  |
|  | % Increase | 50.93 |  |  |  |
| Mississippi | Yes | 1.90 (1.48–2.32) | — | | |
|  | No | 1.26 (0.99–1.53) |  |  |  |
|  | % Increase | 50.61 |  |  |  |
| Nebraska | Yes | 1.88 (1.54–2.21) | — | | |
|  | No | 1.25 (1.03–1.47) |  |  |  |
|  | % Increase | 50 |  |  |  |
| New Mexico | Yes | 1.68 (1.23–2.12) | — | | |
|  | No | 1.18 (0.87–1.49) |  |  |  |
|  | % Increase | 41.82 |  |  |  |
| New York | Yes | 1.31 (0.98–1.64) | — | | |
|  | No | 0.94 (0.71–1.16) |  |  |  |
|  | % Increase | 39.68 |  |  |  |
| Vermont | Yes | 1.82 (1.15–2.50) | — | | |
|  | No | 1.18 (0.70–1.65) |  |  |  |
|  | % Increase | 54.84 |  |  |  |
| Washington | Yes | 1.62 (1.24–1.99) | — | | |
|  | No | 0.94 (0.73–1.16) |  |  |  |
|  | % Increase | 71.39 |  |  |  |

Abbreviations: —, not applicable.

^a^ Age–adjusted to 18–44 y, 45–64 y, 65–74 y, ≥75 y, based on the 2000 census. Percentage increase is the increase due to including repeat hospitalizations.
